# Enhanced Imbibition in Liquid-Infused Coated Microchannels

**DOI:** 10.1021/acs.langmuir.4c03514

**Published:** 2024-12-06

**Authors:** Andreu Benavent-Claró, Sergi Granados Leyva, Ignacio Pagonabarraga, Rodrigo Ledesma-Aguilar, Aurora Hernández-Machado

**Affiliations:** † Departament de Física de la Matèria Condensada, 16724 Universitat de Barcelona, Barcelona 08028, Spain; ‡ Institut de Nanociència i Nanotecnologia, 16724 Universitat de Barcelona, Barcelona 08028, Spain; § Universitat de Barcelona Institute of Complex Systems (UBICS), 16724 Universitat de Barcelona, Barcelona 08028, Spain; ∥ Institute for Multiscale Thermofluids, School of Engineering, 67387 University of Edinburgh, The King’s Buildings, Mayfield Road, Edinburgh EH9 3JL, U.K.

## Abstract

Spontaneous capillary imbibition has the potential to improve the performance of many micro and nanodevices since it does not require an external energy source to drive a fluid flow. Despite this advantage, controlling and reducing the friction exerted by the channel walls, which limits the speed of the liquid, remains a challenge. Here, we demonstrate experimentally that infusing the walls of a channel with a liquid lubricant substantially speeds up the imbibition process and reduces the overall viscous friction. By varying the viscosity of the lubricant, we observe a substantial reduction of the imbibition time of up to 50%. Our experimental results are in good agreement with previous theoretical predictions, providing a solid framework to study low-resistance spontaneous imbibition processes.

## Graphical abstract

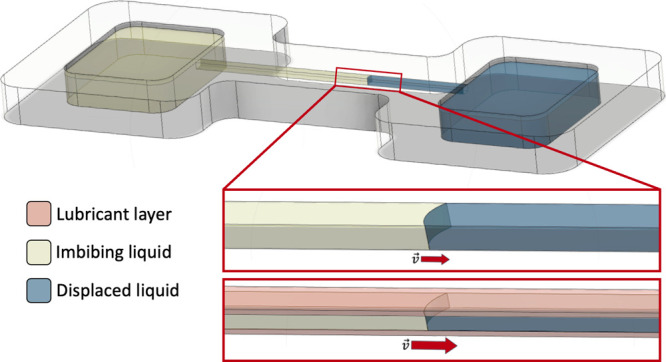

## Introduction

Managing, optimizing, and controlling flows confined in micro and nanofluidic devices is a fundamental challenge relevant to many technological and industrial applications. Over the past decade, substantial improvements to techniques that create micro and nanochannels with controlled surface properties have been made. Such improvements have in turn enabled better strategies to control fluid transport at increasingly smaller scales.^1^

Capillary-driven flows are the most widely used method to drive fluids at micro and nanoscales. Applications can be found in a broad range of fields, including inkjet printing,^2^ lab-on-a-chip experiments,^3^ molecule sensing for point of care diagnostic applications^4^ and soil erosion and flooding.^5^

Mathematically, the canonical model used to predict the motion of the liquid during imbibition is the Lucas-Washburn (LW) law, which is valid for an initially empty channel that is brought into contact with a liquid that has a wetting affinity for it. The liquid will displace the resident fluid (e.g., air) and imbibe the channel.^6^ The invasion takes place spontaneously, and without the need of any external force, which is a key advantage for down-sizing laboratory experiments to small scales. The LW law predicts the penetration length of the liquid, $l∼\sqrt{t}$ , as a function of time *t*, and captures well the late stages of the imbibition process. The diffusive-like dynamics of LW law has been observed in systems such as porous media, like paper,^7^ or other complex textures.^8^ Such slowing-down dynamics is a consequence of the viscous friction caused by direct contact with the solid, which grows with increasing penetration of the liquid into the channel, and represents a major disadvantage to overcome. Furthermore, other factors such as the roughness of the surface can significantly slow down the imbibition process^9^ or even result in pinning of the interface, stopping the flow altogether.

Applications of spontaneous imbibition would benefit from surfaces that are resistant to pinning, while at the same time reduce the imbibition time. A promising solution to achieve these features is to remove the coupling between the solid and the liquid by introducing a lubricant coated surface, such as slippery liquid infused porous surfaces (SLIPS)^10^ or liquid-infused porous surfaces (LIS).^11^ SLIPS and LIS exhibit many interesting properties, such as reduced roughness surface, which results in a small contact angle hysteresis. Besides these properties, SLIPS have been shown to resist extreme conditions, to have self-healing properties and to reduce drag in pressure-gradient driven flows.^12–14^

Recent theoretical and computational results have shown that capillary-driven flows on lubricant-coated surfaces can switch the dissipation from the liquid to the lubricant layer, leading to a faster imbibition of the liquid into the channel.^15^ This effect is similar to the speed-up observed for droplets spreading on liquid-infused surfaces at low viscosity of the liquid lubricant layer.^16^ However, there has been no experimental evidence of the effect of a lubricant layer on fluid dynamics in microchannels.

In this paper, we carry out experimental realizations of spontaneous imbibition in SLIPS coated channels. We show that the SLIPS coating minimizes the roughness of the channel, thus eliminating contact-line pinning. At the same time, the additional lubrication provided by the SLIPS layer reduces the friction with the solid substrate. This friction reduction is due to the fact that the imbibing and displacing fluids are not in contact with the solid wall of the channel, but they are in contact with the liquid layer of the lubricant, reducing the dissipation in both liquids. In the set of experiments performed in this work, the lubricant is less viscous than the displacing phase, leading to a decrease of the dissipation compared to a channel of the same width in the absence of the lubricant.

We found that by choosing the viscosity of the SLIPS coating, the velocity of the front can be increased and the imbibition time can be reduced significantly. We compare our experimental results to the prediction of a previously reported theoretical model^15^ and we obtain a remarkable agreement.

## Materials and Methods

### Microfluidic Setup

A PDMS microchannel was constructed to recreate spontaneous imbibition between two liquids. The schematic representation of the channel is shown in Figure 1A. The channel has a rectangular and constant cross section of width *w* = 350 μm, height *H* = 1 mm, and length *L* = 30 mm. It contains two immiscible liquids, denoted 1 and 2, whose viscosities are η_1_ and η_2_, respectively, and their dynamic contact angle is θ. The walls of the channel are coated with a SLIPS layer, which consists of solid nanoparticles infused with a third immiscible liquid of viscosity η_*S*_. The fraction of the channel occupied by liquids 1 and 2 is *H̅*, so the thickness of the lubricant layer is *h* = (*H* – *H̅*)/2. The channel is connected to two rectangular reservoirs that hold liquids 1 and 2. It is positioned at the top of the reservoirs to prevent hydrostatic pressure from driving the flow.

**Figure 1:**
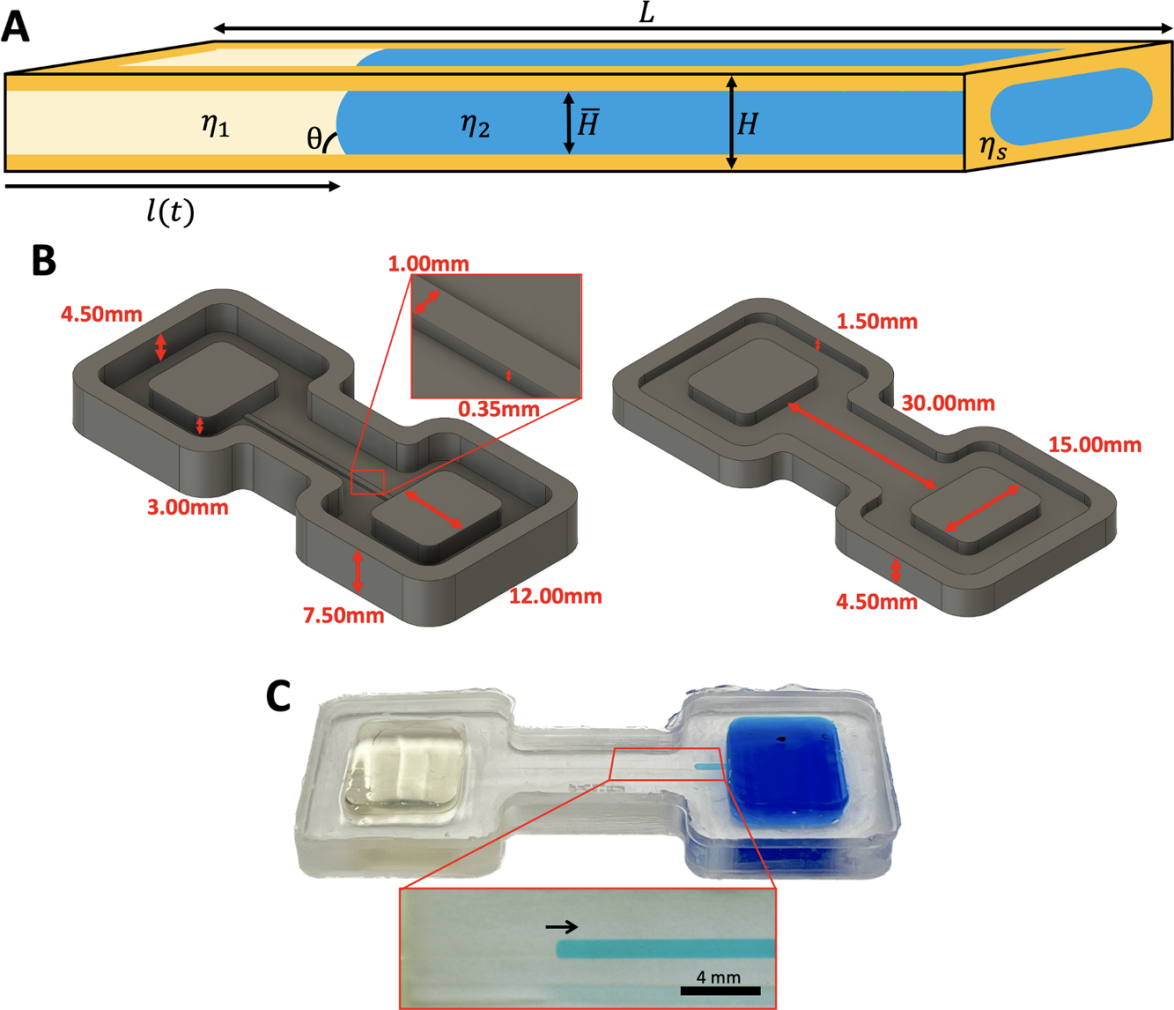
(A) Schematic representation of the system. Liquid on the left (yellow) displaces the liquid on the right (blue) due to capillarity. Contact with the wall is prevented by a SLIPS layer (orange) composed of a nanoparticle–lubricant mixture. (B) Computer design of the 3D negative molds for the experimental microfluidic channel, composed of a base (left) and cover (right). (C) PDMS chip made with the 3D-printed mold of B. The image shows two liquids moving by spontaneous imbibition. Below we show an image taken by the camera for the processing of the data. This particular case corresponds to η_1_ = 1400 mPa s (transparent fluid), η_2_1__ = 480 mPa s (blue fluid), and η_*s*_1__ = 4.8 mPa s.

To manufacture the channels, two negative molds for each chip (base and cover) were designed and produced using a 3D resin printer (Formlabs Form 3+ with Gray Pro V1 resin) (see Figure 1B). Liquid PDMS was poured into the molds and cured for 2 h at 75 °C. The resulting cured PDMS chips were glued and bound under oxygen plasma for 40 s, thus creating the microfluidic setup (Figure 1C). Due to the experimental process the chips are not reusable, so a new chip was created for each experiment.

### SLIPS Coating

To coat the microchannel with a SLIPS coating, we first impregnate all the inner channel walls with nanoparticles and subsequently infuse a liquid lubricant. A volume *V*_G_ = 1.5 μL of a nanoparticle based superhydrophobic coating (Glaco, Soft99) is sprayed into the channel and left to dry for 20 min to create a homogeneous nanoparticle coating. This process is repeated three times so that the different layers of nanoparticles generate a porous structure that will retain the lubricant.^200^

A volume *V*_s_ = 1 μL of silicone oil (Sigma-Aldrich) is then injected into the channel. To obtain a uniform oil distribution, the channel is gently and periodically tilted, so that the oil drop moves from one end of the channel to the other, ensuring a uniform distribution of the lubricant layer. After repeating the tilting process multiple times, the drop is completely absorbed by the channel surface.

### Imbibition Experiments

As a displacing (imbibing) liquid, we use castor oil, which has a viscosity η_1_ = 1400 mPa s. As a displaced liquid, we use two different mixtures of water with glycerol (viscosities η_2_1__ = 480 mPa s and η_2_2__ = 23 mPa s, respectively). Therefore, by adjusting the ratio of water to glycerol in the mixture, we can vary the viscosity of the displaced liquid in the range between the viscosities of glycerol and water. As a lubricant we use silicone oils, which wet the nanoparticle-coated channel preferentially and have a broad range of viscosities. Here, we use silicone oils of viscosities η_*s*_1__ = 4.8 mPa s and η_*s*_2__ = 48 mPa s. All viscosities reported in this study were independently measured using a front microrheometer.^17,18^

The liquids used in this study form an immiscible 3-liquid system,^19^ where castor oil spontaneously displaces the water–glycerol mixture on both an uncoated PDMS channel and in the presence of a SLIPS layer. To visualize the imbibition process, the displacing liquid is dyed in blue (food colorant, Vahine).

Imbibition experiments were carried out by placing the chip on a level stage. The displaced liquid is initially introduced into one of the reservoirs and forced to flow through the channel with a syringe until the channel is completely full. The imbibing fluid is poured into the other reservoir. The imbibition process is recorded using a camera, and the images analyzed to extract the position of the meniscus.

In order to have robust experimental results, each single experiment was repeated three times, each time using a completely new channel and performing a new coating process. This means that, for each data set, there are three completely independent experiments. The experiments between different data sets are also completely independent.

Images of the imbibition process were recorded using a HQ Raspberry Pi camera 12.3 MPx with a Sony IMX477 sensor and a microscopic lens of a focal length of 20 mm. The camera provides a field of view of 29 mm × 22 mm with a resolution of 4032 × 3040 pixels, which allows us to record the front along the whole channel with high resolution. Video recordings were made at 1 fps since the average time from the experiments to move all the channel long is more than 700 s. The video images were processed using the analysis software ImageJ^20^ to measure the position of the front and the dynamic contact angle. These data were then processed using a MATLAB^21^ program.

## Results

Figure 2 shows a comparison of the imbibition time, defined as the time taken for the meniscus to reach 90% of the channel length, for different combinations of the viscosities of the SLIPS layer and the displaced liquid. We tested a total of six configurations based on the inclusion of SLIPS layers, and variations of the viscosities of both, the displaced liquid and the lubricant layer. Remarkably, the effect of the SLIPS coating is always a reduction in the imbibition time relative to the imbibition time in a no-SLIPS (uncoated) channel. Decreasing the lubricant viscosity, η_*s*_, decreases the imbibition time. For the lower lubricant viscosity studied in this work (η_*s*_ = 4.8 mPa s), the imbibition time is reduced by more than half compared to the uncoated channel.

**Figure 2:**
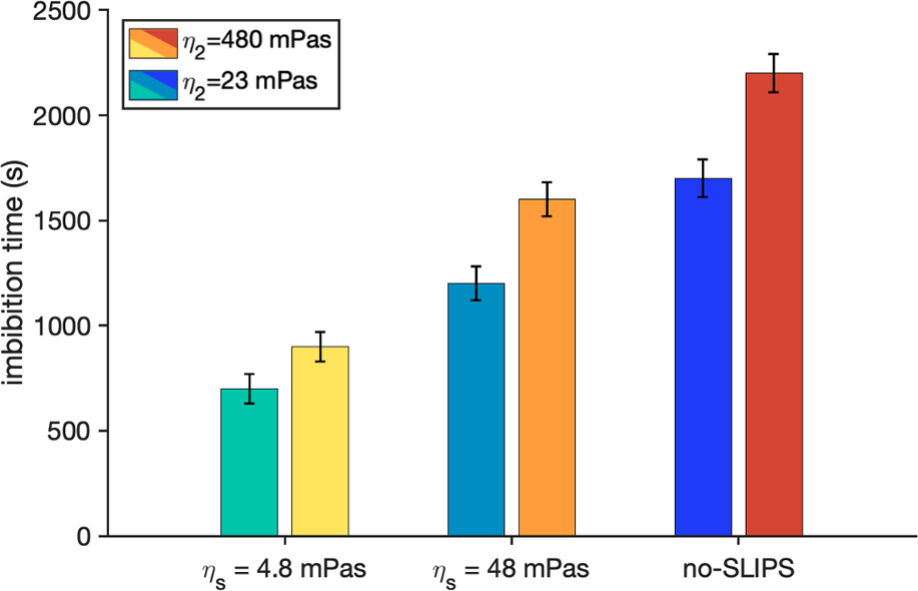
Time required to fill the 90% of the channel as a function of the lubricant viscosity η_*s*_, with their corresponding error-bars. In all cases the viscosity of the imbibing fluid is η_1_ = 1200 mPa s. For the same viscosity of the lubricant layer we have two different viscosities of the displaced liquid η_2_ (see legend).

The imbibition dynamics is driven by capillary pressure, which arises from the curvature of the meniscus and its surface tension. To verify that the observed reduction in the imbibition time is not due to an increase in the capillary pressure, we measured the dynamic contact angle of the meniscus, θ, which determines the interface curvature.

As shown in Figure 3 the dynamic contact angle does not vary significantly upon adding a SLIPS layer, with average values between 40° and 65°.

**Figure 3:**
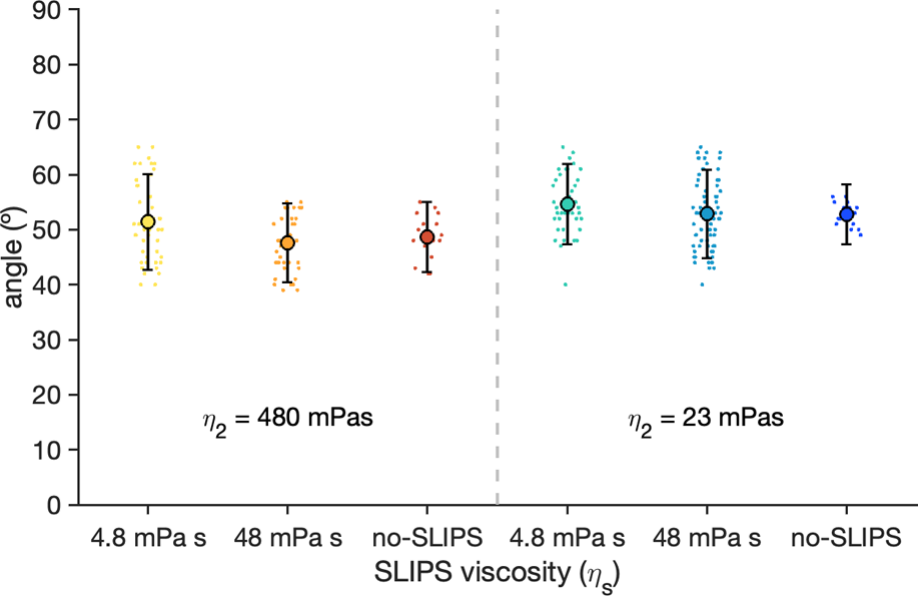
Dynamic contact angle, θ, of the fluid front between castor oil and glycerol/water mixture for each experiment. Point cloud of each experiment corresponds to the measured values, while the thick point corresponds to the mean of the cloud. Error bars correspond to the standard deviation of the cloud.

Such a small variation of the dynamic contact angle supports that the difference in the imbibition time observed in the experiments is not due to changes in the capillary pressure, but due to a reduced friction caused by the SLIPS layer.

To investigate the speed-up caused by the SLIPS coating, we now compare our experimental data with a hydrodynamic model that includes the effect of a thin lubricant layer.^15^ The model is based on the balance of capillary and viscous friction forces, and neglects the effect of inertia on the liquids. The lubricant layer is assumed to be of uniform thickness, and completely adhered to the walls of the channel. We assume that the SLIPS lubricant has zero mean velocity because the wicking properties of the surface create capillary pressure which counteracts the shear flow of the imbibing fluids, stabilizing the lubricant layer.^15,22^ Given the typical velocities and surface tensions in this work, the capillary number is small Ca = *v*_0_η/γ ∼ 10^–4^, and we expect the lubricant thickness to remain constant during the imbibition. The velocities at which the fluids move are not high enough to significantly move the lubricant, and we do not expect entrainment to occur. Under these considerations, the force balance results in a prediction of the position of the meniscus, *l*(*t*)/*L*, as a function of time, *t*, and reads^15^

$$\frac{\lambda -1}{2}\frac{l^{2}(t)}{L^{2}}+\frac{l(t)}{L}=\frac{\Delta p\overset{®}{H}\lambda (3h\eta_{1}+2\overset{®}{H}\eta_{s})}{24L^{2}\eta_{s}\eta_{1}}t$$ 1

where Δ*p* = γcosθ/*H* is the capillary pressure, γ is the surface tension between the liquids; *h* is the height of the lubricant layer and

$$\lambda =\frac{\eta_{1}}{\eta_{2}}\frac{3\eta_{2}h+2\overset{®}{H}\eta_{s}}{3\eta_{1}h+2\overset{®}{H}\eta_{s}}$$ 2

quantifies the effect of the viscosities of the three liquids and the relative size of the SLIPS layer to the thickness occupied by the displacing and displaced liquids.

The magnitude of the pressure drop is determined by the dynamic contact angle, θ, that includes the effect of the meniscus friction force.^15^

The dynamics of imbibition is determined by the interplay between the two terms on the left-hand side of eq 1. At short times, the linear term dominates and thus the equation reduces to the scaling *l*(*t*) ∼ *t*. At long times, the quadratic term dominates and one recovers the LW scaling *l*(*t*) ∼ *t* ^1/2^. Crucially, the crossover between these regimes is controlled by the viscosity ratio through λ. When λ → 1, the dynamics of the fluid front are linear in time. This behavior can come from two different effects: On one hand, when the imbibe and the displaced fluids have the same viscosity (η_1_ = η_2_), the dissipation generated in the channel is always constant. On the other hand, this linear regime is also achieved when the viscosity of the lubricant is very low (η_*s*_ ≪ 1). In this case, all the dissipation is due to the lubricant layer of constant size, which gives a constant velocity. In this limit, eq 1 reduces to

$$l(t)=\frac{\Delta p\overset{®}{H}(3h\eta_{1}+2\overset{®}{H}\eta_{s})}{24L\eta_{s}\eta_{1}}t$$ 3

The other limiting case is found when λ ≫ 1, in this case, we recover the behavior of LW equation. This behavior occurs when the viscosity of the displaced liquid is negligible with respect to the imbibing liquid η_1_ ≫ η_2_. In this limit, the viscous fluid occupies a larger fraction of the channel as a function of time and eq 1 reduces to

$$l(t)={\left(\frac{\Delta p\overset{®}{H}(3h\eta_{1}+2\overset{®}{H}\eta_{s})}{12\eta_{s}\eta_{1}}\right)}^{1/2}t^{1/2}$$ 4

A third limit arises when there is no lubricant layer in the channel. In this limit, *h* = 0. Then, eq 1 reduces to the capillary imbibition equation for two viscous fluids^23,24^ :

$$\frac{1}{2}\left(\frac{\eta_{1}}{\eta_{2}}-1\right)\frac{l^{2}\left(t\right)}{L^{2}}+\frac{l\left(t\right)}{L}=\frac{\Delta pH^{2}}{12\eta_{2}L^{2}}t$$ 5

To compare the theory with our experimental results, we fitted eq 1 to the experimental data. In the model, all parameters are known except for the interfacial tension between the displacing and the displaced liquids and the effective thickness of the SLIPS layer. Therefore, we treated these parameters as fitting constants, which we discuss below.

The interfacial tension can be estimated using Antonov’s rule, which states that the surface tension between two liquids is approximately equal to the difference in surface tensions between the liquids and air: γ_AB_ = |γ_A_ – γ_B_|.^25^ Castor oil has a surface tension of approximately 39 mN/m.^26^ Due to the purity of the sample, in our case, this value may change to γ_1_ = (39 ± 2) mN/m. On the other hand, glycerol mixtures with a high fraction of glycerol have a surface tension of approximately 65 mN/m.^27^ In our case, due the difference of concentration we add an error to the value: γ_2_ = (64 ± 4) mN/m. Using Antonov’s rule, the surface tension is γ ≈ |γ_1_ – γ_2_| = (25 ± 6) mN/m. This value matches the values obtained by fitting the model to the experimental data, leading to small deviations of a few mN/m, all of which fall within the expected error of the estimate. We do not expect the silicone oil lubricant to get between the castor-glycerol interface. Using Antonov’s rule, and considering the surface tension of silicone oil (γ_*s*_ = 21 mN/m), we find that the energy required to form the two new interfaces (γ = 61 mN/m) is significantly higher than the energy associated with the moving fluid interface (γ = (25 ± 6) mN/m).

Regarding the effective thickness of the SLIPS layer, we note that eq 1 is derived assuming a liquid lubricant layer, and not a composite SLIPS layer formed by nanoparticles and a lubricant. Therefore, we can only estimate an upper bound for the lubricant layer thickness.

Although the use of GLACO and silicone oil to create SLIPS in a closed channel, as done in this study, has not been previously reported, it has been done in open geometries, resulting in a lubricant layer thickness ranging between 1 and 10 μm.^13,28^ We assume that the lubricant is uniformly distributed in all four internal surfaces of the channel. The total internal surface area of the channel is *S*_ch_ = (2*H* + 2*w*)*L*, and the volume of silicone oil used is *V*_s_ = 1 μL. Using these values, we obtain *h* ≈ *V*_s_/*S*_ch_ = 12 μm as an upper bound. Although we have not been able to measure the thickness of the lubricant layer directly, it can be estimated by fitting eq 1 to the experimental data. We consistently obtain values of the SLIPS layer thickness *h* ≃ 1 μm. The consistent and substantial increase in the dynamic speed of the fluid front provides additional evidence of the presence of the lubricant layer.

Figure 4 shows the experimental $\frac{l\left(t\right)}{L}$ vs *t* curves for the same combinations of viscosities of Figure 2.

**Figure 4:**
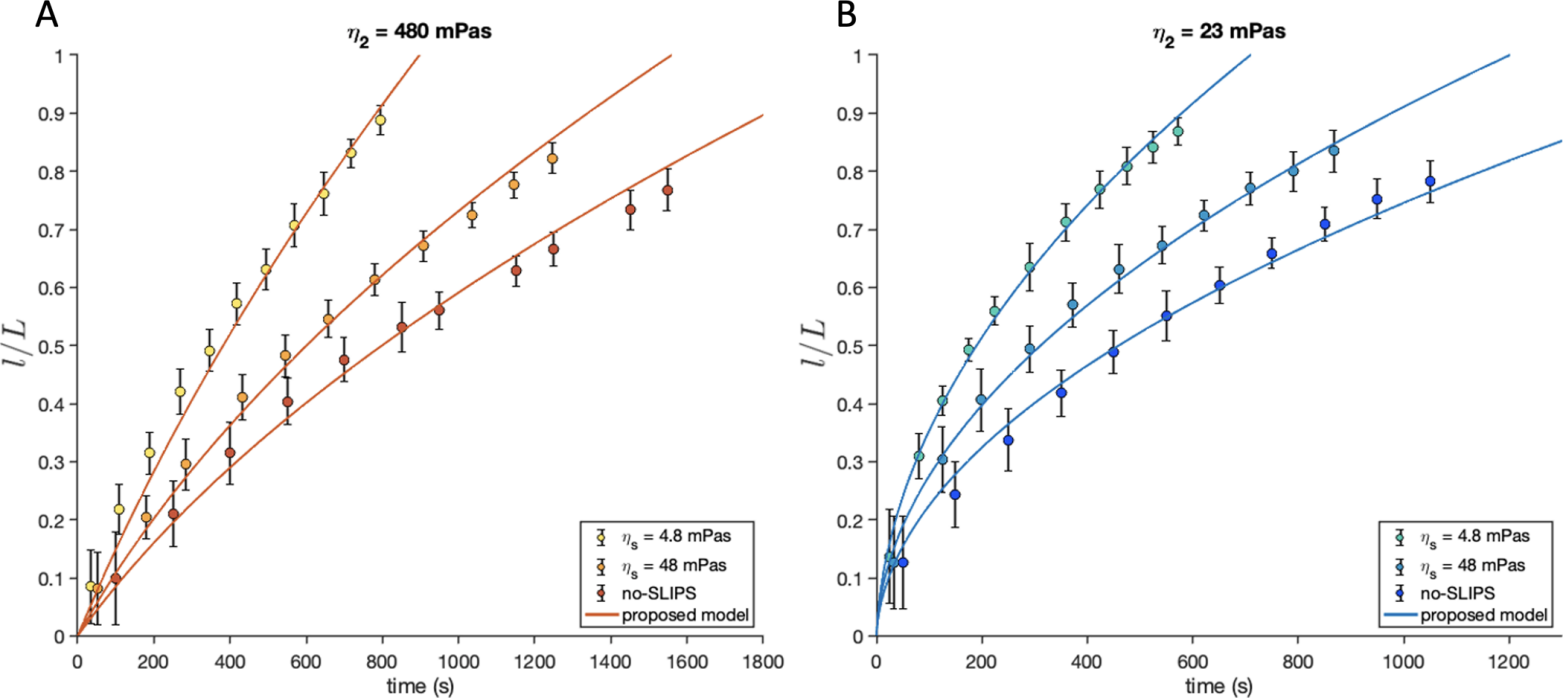
Normalized front position as a function of time for (A): η_2_ = 480 mPa s and (B): η_2_ = 23 mPa s. In all cases the imbibing liquid has a viscosity of η_1_ = 1400 mPa s. Each data set corresponds to a different channel coating: SLIPS with η_*S*_ = 4.8 mPa s (light-colored markers), SLIPS with η_*S*_ = 48 mPa s (medium light-colored markers), and without SLIPS (dark-colored markers). Solid lines corresponds to fits the theoretical model, eq 1.

The data clearly show that for a fixed invading/displaced viscosity ratio, decreasing the lubricant viscosity speeds-up the imbibition process, allowing the front to reach the end of the channel sooner. In this experiment, we can observe that, by changing the lubricant to one that is ten times less viscous, results in a reduction of the imbibition time by more than half. This imbibition speed-up is in agreement with the theoretical model, which can quantitatively capture the experimental results.

We can express eq 1 in dimensionless units, leading to a new eq 6 which shows that the kinetics of the imbibition follows a universal curve.

$$\frac{1}{2}{\hat{l}}^{2}(t)+\hat{l}(t)=\hat{t}$$ 6

where

$$\hat{l}=(\lambda -1)l(t)/L$$ 7

and

t^=ΔpH¯(λ−1)λ(3hη1+2H¯ηs)24L2ηsη1t 8

This universal equation is characterized by a linear dependence at short normalized times (eq 3) and LW law asymptotically at long normalized times (eq 4). The crossover time between these two regimes takes place at t̂* = 2, which corresponds to t* = 48 *L* ^2^ η_*s*_ η_1_/(Δ*pH̅*(λ – 1)­λ­(3*h*η_1_ + 2*H̅*η_*s*_)). This crossover time, t*, moves to longer times when the viscosity of the displaced liquid increases, implying that the linear growth will persist longer. Likewise, decreasing the lubricant viscosity also delays the crossover to the LW regime. Figure 5 represents this normalized position as a function of normalized time, where we can clearly see this behavior. In the absence of a SLIPS layer, the growth exponent increases from *n* = 1/2 to *n* = 1 as the viscosity of the displaced liquid varies from η_2_ = 0 to η_2_ = η_1_. This is due to an increased viscous friction in the displaced phase.^23^ The effect of the SLIPS layer is to shift the crossover between the linear and square root behavior to higher values, so that its representation in the normalized graph is always more shifted to the linear behavior than the uncoated channel. This shift is stronger for lower viscosity of the lubricant layer, and for intermediate values of the displaced liquid, which corresponds to the regime studied in this paper.

**Figure 5:**
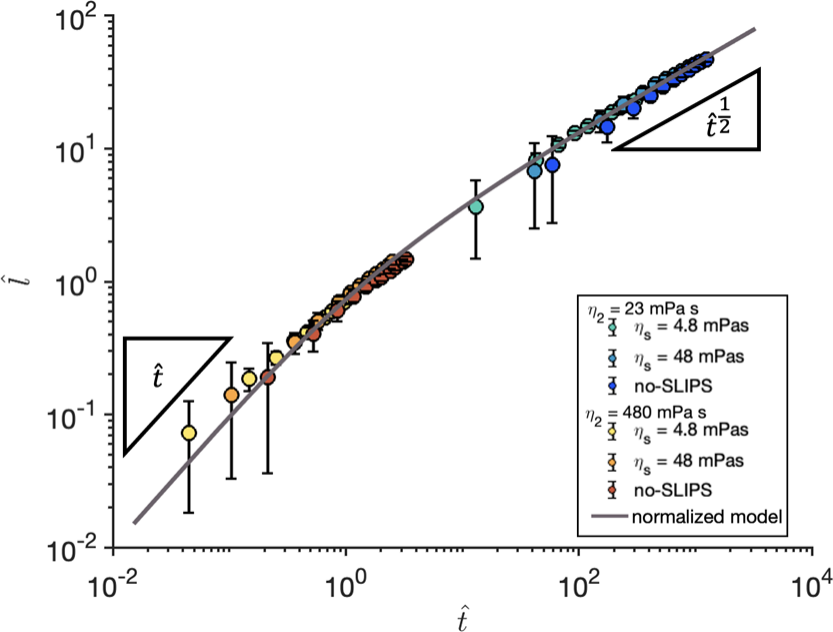
*l̂* given by eq 7 as a function of *t̂* given by eq 8 in log–log scale for all experimental data sets. It includes the representation of the slopes of the limit cases *t̂* and *t̂* ^1/2^ and a general curve given by eq 6.

## Conclusions

In this paper, we have studied the spontaneous imbibition of a liquid in a microchannel with walls made of slippery liquid infused porous surfaces (SLIPS). Our results show that the imbibition process occurs faster in channels coated with SLIPS walls, and that the imbibition time decreases significantly as we lower the viscosity of the SLIPS layer. The LW equation for two fluids is not satisfied when the channel has a SLIPS coating. Instead, the experimental data is well described by the model presented in ref 15, which includes the effect of a liquid lubricant layer.

One may wonder if the increase in imbibition speed caused by the SLIPS layer comes at a cost in the viscous friction resisting the flow in the microchannel. Following ref 15, the friction force per unit length of the front is given by eq 9

$$F_{\eta }=\frac{8\langle v\rangle \eta_{s}L}{h}\left(\frac{\frac{l(t)}{L}}{1+\frac{2\mathrm{H̅}\eta_{s}}{3\eta_{1}h}}+\frac{1-\frac{l(t)}{L}}{1+\frac{2\mathrm{H̅}\eta_{s}}{3\eta_{2}h}}\right)$$ 9

where ⟨*v*⟩ is the mean velocity of fluid front. In our experiments, the largest speed up of the front occurs for a SLIPS layer of viscosity η_2_ = 23 mPa s and a viscosity of the displaced liquid η_*s*_ = 4.8 mPa s. Comparing this case to an uncoated channel gives a reduction in viscous friction of 53%. Therefore, the increase in imbibition speed comes with a reduction (and not an increase) in viscous friction.

Increasing the rate of spontaneous imbibition within a microchannel without altering the fluids involved brings multiple technological advantages. The main advantage of this strategy lies in the rapid filling of microchannels, which significantly reduces the time required for fluid imbibition. Such a speed-up increases the responsiveness of microfluidic systems, and can help to provide rapid results in a variety of applications, such as disease diagnosis and environmental monitoring. A higher imbibition velocity not only optimizes fluid transport, minimizing the risk of fluid entrapment, but also promotes more precise and controlled flow. Consequently, with this method, lab-on-a-chip devices could improve their performance, reducing experimentation time. This optimization promises efficiency, responsiveness and higher throughput in various microfluidic applications, establishing a versatile and high-impact strategy.
